# Indoor mould testing in a historic building: Blickling Hall

**DOI:** 10.1186/s40494-018-0218-x

**Published:** 2018-08-30

**Authors:** Yasemin Didem Aktas, Jiaqi Shi, Nigel Blades, Dina D’Ayala

**Affiliations:** 1 University College London (UCL) Department of Civil, Environmental and Geomatic Engineering, Epicentre Research Group, Chadwick Building, Gower Street, London, WC1E 6BT UK; 2 UK Centre for Moisture in Buildings, London, UK; 3 National Trust for England, Wales, Northern Ireland, Norfolk, UK

**Keywords:** Indoor mould growth, Active (aggressive) sampling, Air sampling, Surface sampling, NAHA

## Abstract

Indoor mould growth is a growing concern for all stakeholders of built environment, including residents, builders, insurance and building remediation industry as well as custodians of heritage buildings. The National Trust has reported this problem in a number of buildings under their ownership, and developed solutions and fine-tuned their maintenance programme so as to minimise indoor and surface mould growth risk. This paper reports findings from an extensive mould-testing scheme in Blickling Hall, a National Trust property in Norfolk, England, for an appraisal of airborne and surface mould levels within a total of eight rooms, including the famous Long Gallery. The testing protocol used combines active (aggressive) air sampling and surface sampling, analysis of the β-*N*-acetylhexosaminidase (NAHA) activity to quantify mould levels and particle counting. The results show that the airborne mould levels are quite low in all spaces, due to satisfactory maintenance of indoor hygrothermal conditions by conservation heating. On the other hand, while the National Trust’s developed solutions and maintenance programme have proved effective to avoid surface mould growth in those locations that historically suffered from microbial activity (such as behind book presses, picture frames and tapestries), the results show that the surface cleaning around windows should be improved to tackle surface water due to condensation, which is considered to be the main driver behind high surface NAHA activity obtained in these areas.

## Introduction

Indoor mould growth is one of the most common factors that cause adverse health effects and fabric deterioration in buildings [1–3]. Historic buildings may be more prone to material decay and other physical/mechanical processes caused by mould growth because of accumulated material damage, exacerbated in certain cases by poor maintenance, drainage issues, poor hygrothermal performance, water leaks and water infiltration into the building envelop due to driven rain or other weather events [4].

Mould growth is a complex process, facilitated by the co-presence of certain hygrothermal conditions, often defined in the form of species-/substrate specific temperature and relative humidity isopleths [5], which, if no other data are available, are frequently used for the appraisal of an indoor environment’s susceptibility to mould growth (e.g. [6, 7]). Other methods of assessing the mould growth potential include performance models based on dose–response functions developed for certain materials [8–10]. A more detailed analysis of mould presence and intensity in the air or on a surface of a given indoor environment, however, requires direct testing [11]. There are many analytical techniques that can be used to measure mould levels, including culture-based, microscopic, chemical and immunoassay methods, and more recently, polymerase chain reaction (PCR) based methods, each with their respective strengths and biases (see [12] for a review of various techniques). In this study we apply a chemical method which uses the quantification of *N*-acetylhexosaminidase (NAHA) activity to measure mould levels. NAHA has been found to be a reliable indicator of mould cell biomass, with high sensitivity and specificity [13–17]. The method allows both for surface and air examination, and was previously shown to be effective in the investigation of mould levels within heritage buildings [18].

**Fig. 1:**
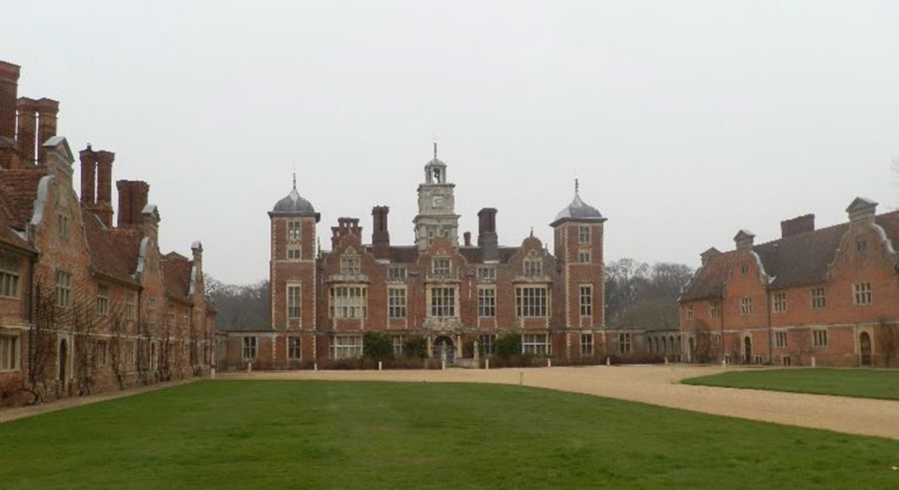
Blickling Hall

The case study building here is the early seventeenth century Blickling Hall, a Grade I listed National Trust property in Norfolk, England (Fig. 1), built within an approximately 340 ha park and well-known for its extraordinary architectural features and its Jacobean Long Gallery, housing one of the most significant National Trust libraries [19]. This remarkable building, located within the River Bure basin, can flood during episodes of heavy rainfall and is subject to wind-driven rain, which, combined with past failures of rainwater goods, have encouraged water-induced problems within the building, including dampness and biodeterioration in the basement, and mould growth in the library and other showrooms [20, 21].

Previously, a rigorous environmental monitoring work was carried out in Blickling Hall for an assessment of the hygrothermal performance of the building envelop [25]. This showed critical moisture enrichment within the outer walls under wind-driven rain exposure. This study also assessed the mould growth susceptibility in a number of locations within the Hall using isopleths developed for Substrate Category I, and concluded that while in the basement mould growth was a realistic threat (in broad agreement with the past and current condition of the basement), the Long Gallery indoor conditions did not suggest mould risk. In the present study, we report our findings from an air and surface mould-testing campaign carried out in February 2017 in eight rooms at Blickling Hall, including the famous Long Gallery, for the aim of an appraisal of mould levels within the Hall, as well as the cleaning and maintenance programme of the National Trust.

## Prior incidents of mould growth and attempts at their mitigation

**Fig. 2:**
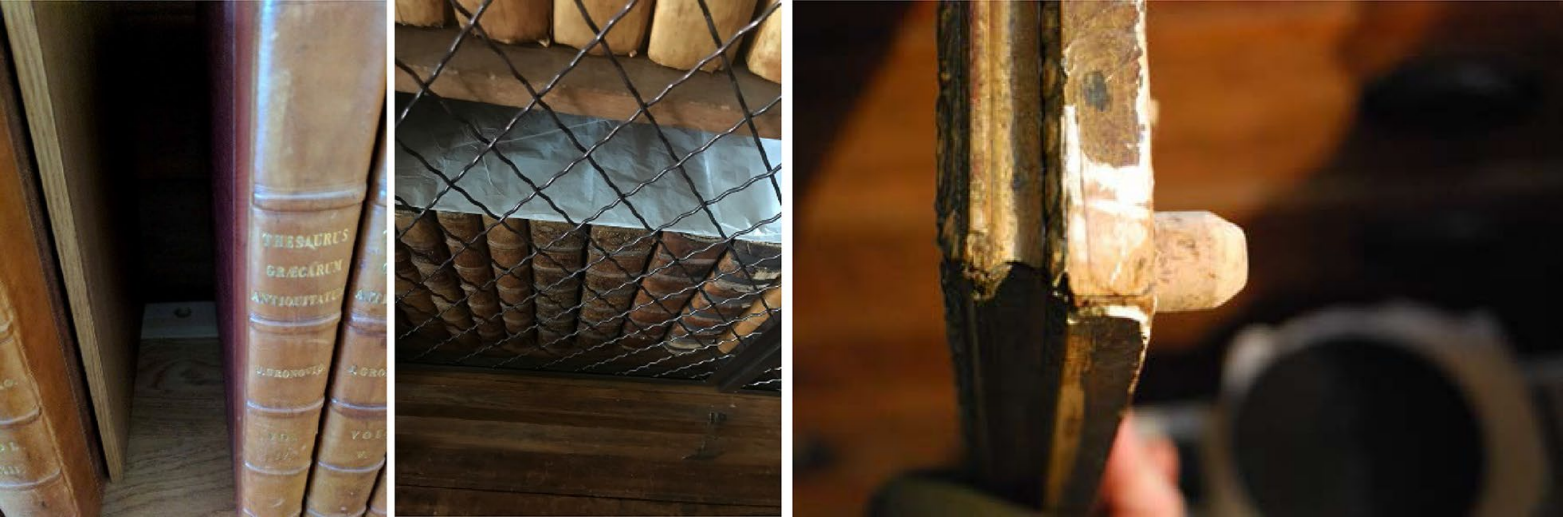
Various means developed by National Trust to minimise odds for mould growth. Left: new timber insert at back of shelf with ventilation hole; middle: taffeta cover protecting text blocks from dust accumulation and resultant mould growth; right (© Andrew Bush/National Trust): cork spacer to support frames so that they are not flush with the wall

Most of the surface mould growth historically reported in Blickling Hall occurred behind tapestries, on the back of paintings and behind book presses within the Long Gallery and Upper Ante-Room, where air circulation is low and temperature is lower due to heat loss through the external walls. The National Trust has tried to tackle the mould growth induced by stagnation due to low ventilation in these areas in various ways: the depth of the bookshelves in the Long Gallery was increased by the insertion of new timber elements in the 1980s into which ventilation holes were drilled at the back of the book presses. The book presses were moved out slightly from the external walls to provide a bigger air gap between wall and presses. Further, the books have been covered with silk taffeta to minimise the dust accumulation which can facilitate microbial growth, while maximising breathability. In order to encourage ventilation behind picture frames, bottle corks have been placed in the transversal direction behind the frames to support them so that they are not flush with the wall (Fig. 2).

Another important factor that makes Blickling Hall, and historic museum/art gallery indoor environments in general, more susceptible to mould growth is the presence of abundant organic materials, such as paper, parchment, textile and leather, which provide nutrients for mould growth. In conjunction with low ventilation, this explains why surface mould growth has historically been concentrated at the back of vellum-covered books, silken wall covers, and tapestry, where, under suitable conditions, fungi readily grow, resulting in damage ranging from stains to complete decay [22, 23]. The two strategies, suggested particularly for the historic museum/art gallery indoor environments, are therefore to keep the RH under 65%, and keep surfaces clean and free of dust at all times [23]. To maintain a suitable environment, the National Trust stabilises indoor hygrothermal conditions at moderate relative humidity and temperature levels using humidistatically controlled conservation heating. In this approach space heating operates when the RH is above a set point of 58–60% and is switched off when the RH falls below the set point, so that a moderate RH can be maintained that is below the threshold for mould growth but not so low as to risk drying and shrinkage of hygroscopic materials [24].

Another location within the Hall that has suffered from heavy mould growth is the basement. This space, closed to public as it does not house any collections hence not benefitting from conservation heating, is buried within saturated soil below the window level, leading to sustained high levels of moisture within the fabric. Combined with faulty drainage, heavy mould growth was recorded in the basement as early as late nineteenth century [20], which led to the tanking of the space. In the early 2000s, the drainage was further improved to ensure effective surface water management in the face of meadows inundating the front forecourt of the house. The space however is still covered with what is considered heavy microbial growth.

## Description of tested rooms and methodology

In this study, eight rooms within Blickling Hall were tested: Long Gallery, Upper Ante-Room, Peter the Great Room, State Bedroom, Brown Drawing Room, Lower Ante-Room, and the basement, which are known to have suffered from surface mould growth at some point, and the Chinese Bedroom, where mould growth has never been spotted, as a control case (Table 1).

**Table 1:** Tested rooms with their corresponding history of mould growth

Room		History of visible surface mould growth
Long Gallery		At the rear of book stacks on, leather and vellum book bindings, and at the sides of the fireplace
Upper Ante-Room		Behind the tapestries and at the rear of the book presses
Peter the Great		On the silken wall cover and behind the picture frames
State Bedroom		Under the bed
Brown Drawing Room		Behind the picture frames
Lower Ante-Room		Behind the tapestries and picture frames
Chinese Bedroom		None
Basement^*^		On most visible surfaces

*Basement was not air-sampled due to lack of a working power source to connect the air pump

The testing protocol employed in this study was composed of the following steps: (a) surface sampling, (b) active air sampling and (c) active particle counting.

Surface sampling was performed to investigate whether there were any localised surface mould problems within the tested rooms, and to check if the previously reported surface mould issues had been resolved. In this study, swabbing, which is one of the most common surface sampling technique [12], was used. Each sampled area, delineated using a 3 × 3 cm adhesive template, was swabbed by means of sterile cotton swabs. As such, 6–14 samples were collected in each room, from locations where surface mould was previously reported, areas that were thought might be suffering from condensation or relatively poor cleaning, and from other surfaces to ensure a relatively even distribution of sampling. The collected samples were tested for the activity of β-*N*-acetylhexosaminidase (NAHA): to the swabs were added an enzyme–substrate containing 4-methylumbelliferyl, and after a reaction time (around half an hour, depending on the temperature), the resulting fluorescence was measured using a hand held fluorometer (Turner Design US/Mycometer version) in relative fluorescence units, RFU (one RFU is equal to 33.3 × 10^−2^ pmol 4-MU per mL reaction volume per min), and substrate blank value was subtracted.

Air sampling is used to quantify airborne mould concentrations and, depending on the technique used, to examine species composition, especially in studies focussing on health implications, as it provides a better understanding of airway exposure than surface testing [12]. Air can be sampled passively (non-aggressively) and actively (aggressively), i.e. from the still air and from the actively mixed air, respectively. Passive sampling detects the fungal material that is readily airborne. The readings obtained by means of passive sampling are therefore very much dependent on the level of activity that has taken place within the tested space right before the testing. When no prior activity is allowed within the space to be tested for control purposes, passive sampling is known to potentially lead to underestimation of mould concentrations within a given indoor environment, as it cannot detect “table-top”, i.e. settled dust on surfaces. Active sampling, on the other hand, uses active mixing of the air in the room to be tested-it will suspend settled dust so its mould activity can be characterised, and has previously been shown to be strongly correlated to the presence of visible mould and other moisture induced problems within the space [11]. In this study, an active air sampling strategy was adopted for its ability to rule out the impact of the prior disturbance (or lack of it) within the space on the readings [26–28]. To this end, the air within each room was actively mixed using a Makita blower to mimic some level of activity, standardised by means of blowing duration based on room sizes as follows: 1 min for room sizes up to 10 m^2^, 2 min for room sizes up to 20 m^2^, 3 min for room sizes up to 30 m^2^, and 4 min for larger room sizes. Locations that are rarely cleaned and are therefore likely to be large mould reservoirs were not blown air in order to avoid pushing the mimicked activity levels beyond what is realistic for Blickling Hall: groups of people walking around while visiting the rooms or cleaning by National Trust staff. Sampling was started after 1 min following the blowing (to allow very large particles to settle), and was made on a MCE-membrane filter (pore size 0.8 µm) using a flow rate of 15 L/min, and the samples were tested for the activity of β-*N*-acetylhexosaminidase (NAHA) in the same way explained above. Each room was sampled for 15 min, apart from the basement, which could not be air-tested due to lack of a working plug to connect the air pump.

Finally, particle counting was done using a CEM Particle Counter (Model DT-9880; flow rate 2.83 L/min with six channels: 0.3, 0.5, 1.0, 2.5, 5.0 and 10 µm). This device was additionally used to record temperature (T) and relative humidity (RH) values at the time of testing. Particle counting was also done actively, and was started simultaneously with the air mould sampling. The only exception to this is the basement, where the particle count was measured only passively, i.e. from still air. The reason for this was higher dust levels and visual evidence for heavy microbial contamination within the room, which, if the air is actively mixed, was considered that might lead to heavy exposure to dust and fungal particles in spite of the facemask that was used during all testing.

## Results and discussion

The NAHA activity values (known also as Mycometer values) (Table 2) show that all rooms tested in this study had very low airborne mould concentrations. According to the benchmarks recently developed for the UK building stock and for the same testing protocol as the one used here, NAHA activity values above 1700 RFU could be indicative of mould growth and therefore require further investigation of the space for a mould source [26]. While left unmeasured, the visual evidence suggests that basement should exhibit much higher airborne mould concentrations. However, the testing results show that all of the rooms that house valuable collections and are maintained in line with National Trust’s cleaning programme have NAHA activity levels below 475 RFU, which is a sign of excellent housekeeping. The comparatively higher NAHA activity obtained from the Brown Drawing Room is most likely due to the presence of more upholstery compared to other rooms, which can potentially act as dust and mould depository—the relatively high particle count (highest in the dataset, both in terms of the total particle count and the PM2.5) supports this conclusion. As a matter of fact, the Brown Drawing Room is the only room where the furniture and chairs were not covered at the time of testing.

**Table 2:** Summary of air sampling results: air mould concentrations (NAHA activity in RFU), temperature (T) and relative humidity (RH) at the time of testing (°C and %, respectively) and particle counts (for each individual channel and total value)

Room	NAHA activity (RFU)	T (°C)	RH (%)	Particle count (Actively measured for all rooms except for basement)						
				0.3 μm	0.5 μm	1.0 μm	2.5 μm	5.0 μm	10 μm	Total
Long Gallery	9	13.4	61.6	212,218	64,197	8440	850	127	77	285,909
Upper Ante-Room	36	13.0	63.4	216,647	63,924	7824	782	115	69	289,361
Peter the Great Room	120	13.6	56.0	218,315	62,659	8313	890	138	86	290,401
State Bedroom	124	13.6	55.3	222,814	70,078	9158	978	132	121	303,281
Brown Drawing Room	475	14.3	57.9	253,258	80,414	11,407	1456	215	297	347,047
Lower Ante-Room	111	15.1	57.3	225,425	72,185	10,676	1391	244	275	310,196
Chinese Bedroom	62	13.2	59.0	203,921	52,282	7212	784	118	110	264,427
Basement	N/A	15.1	53.9	491,232	161,517	19,428	1770	187	81	674,215

The T and RH values at the time of testing vary between 13–15.1 °C and 53.9–61.6%, respectively, and indicate a cool/dry and stable indoor environment throughout the Hall, including the basement. While these values are spot readings and they do not inherently give the long term hygrothermal performance of the Hall indoor environment, the conclusion regarding stable and low T and RH conditions is in broad agreement with the findings of the 2-year monitoring scheme that was previously carried out in the Hall [25]. This previous study had shown that the National Trust’s conservation heating strategy worked well to avoid both all peaks of very high RH that initiate spore germination and prolonged periods of elevated RH that support mould growth, which are the two critical conditions to be controlled to rule out mould growth [23].

The particle counts obtained from the basement indicate a striking difference from the other rooms—although the particle count was taken only passively in the basement, i.e. from still air, the sum of all six channels is still almost two times the highest particle concentration measured in the other rooms. A previous study conducted using the same testing methodology found out that in particle intensive spaces with visible mould the actively measured particle counts could be > nine times higher than passively measured particle counts [11], therefore it can be argued that the particle intensity of the basement could be significantly more than twice as much. There are mixed findings as for the correlation between particle counts and indoor mould concentrations ([29, 30], cf. e.g. [31]), however it is known that under suitable conditions dust within a given space can provide microbial agents with nutrition they need. Therefore, despite the fact that the basement does not house any valuable collections, it would be beneficial to keep this space also clean to minimise the risk of material degradation to the building envelop due to biodeterioration. Importantly, Chinese Room was found to be the least particle intensive of all tested spaces. This room also led to the third lowest NAHA Activity value, therefore proved to be a good control for this study.

**Fig. 3:**
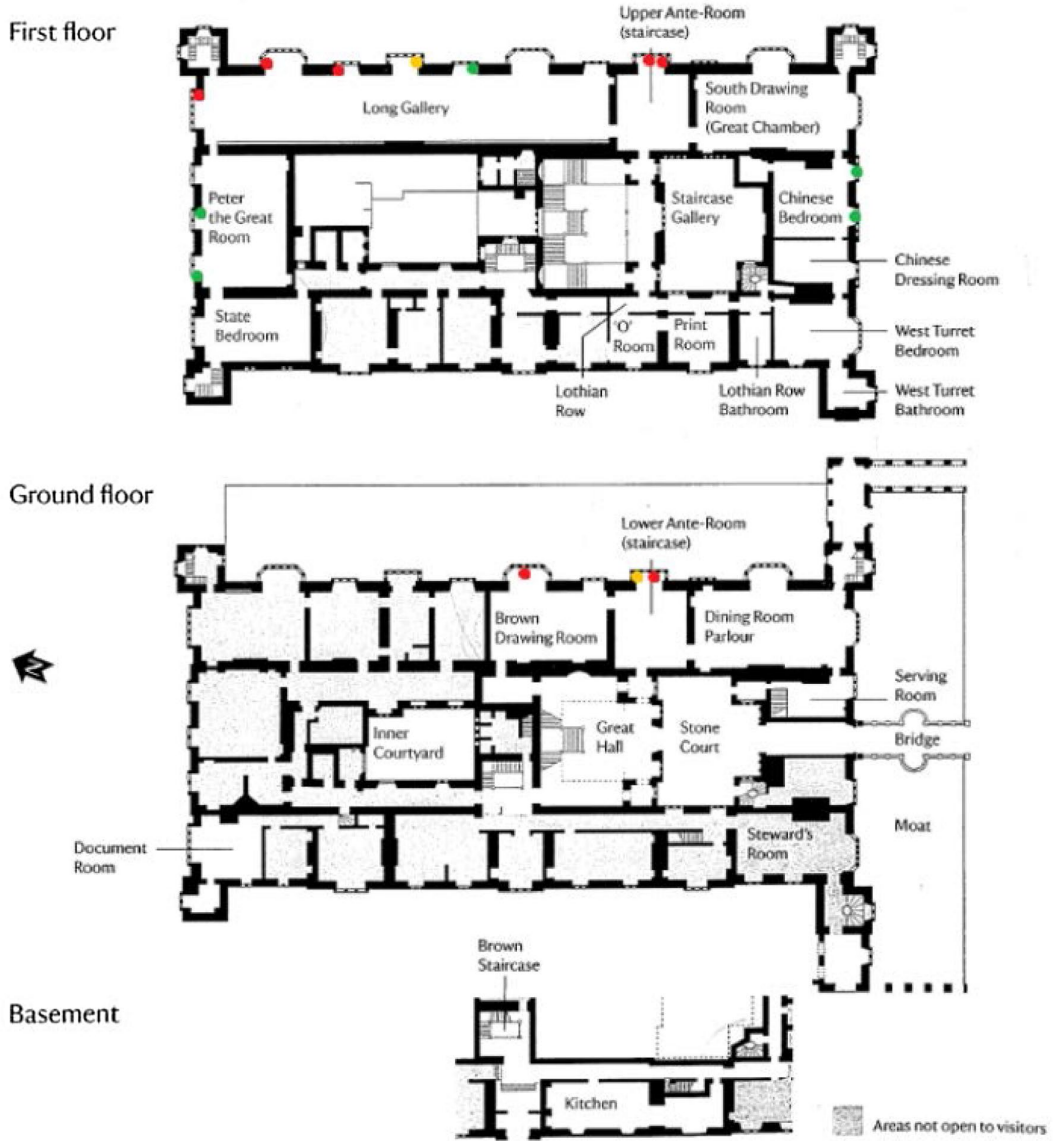
Summary of the results of all surface sampling from around windows (green, yellow and red dots represent surface readings < 25 RFU, 26–450 RFU and > 450 RFU, respectively) (Plan drawing © [33])

An analysis of surface samples led to mixed findings. The majority of the surface samples (55 out of the total of 71 surface samples collected from all rooms) were found to have NAHA activity below 25 RFU, which was suggested to be the upper threshold defining clean surfaces with no mould growth at all [26]. All locations with past mould problems, such as the vellum binders, back of the book presses, fireplaces, silken wall covers, behind the picture frames and tapestries, were found to have very low NAHA activity values, below 50 RFU: according to the abovementioned benchmarks developed for the UK properties, NAHA activity between 26 and 450 RFU indicate surfaces with a poor cleaning standard (the dust and dirt deposits elevate the NAHA activity), but devoid of active mould growth, while values beyond 450 RFU would suggest serious mould growth potential (see [26] for more details). This, once again, shows the effectiveness of National Trust’s cleaning programme and points out the importance of conservation heating for environmental control and keeping surfaces clean. However, importantly, all samples that were found to have NAHA activity above 450 RFU came from around the windows (Fig. 3), with the only exception being samples taken from the light green mouldy surfaces in the lower elevations in the basement. This shows poor hygric performance of the windows, especially those on the southeast facing façade which had previously been shown to be affected by wind-driven rain. This leads to surface water manifesting as a result of condensation, especially in winter months. Mould still germinates and grows on building materials under very low air humidity when water is available on the surface, where the surface RH has arrived 100% [32]. This makes condensation an important indicator of potential mould growth areas, in this case, around window frames, specifically when they are aged and not completely sealed, as here. It is therefore of great importance that these areas are cleaned often and regularly to remove the surface water.

Importantly, the surface samples taken from microbial growth in the basement manifesting itself in dark green did not indicate mould growth, with NAHA activity levels around 100 RFU. To further investigate the lower-than-expected NAHA activity associated with these samples, an additional microscopy analysis was performed, which revealed that this was algal growth, encouraged by sunlight reflecting on the wall through the window opposite. Algal biofilms are also indicative of excess moisture and cause deterioration, but more importantly increase retention of water via soiling in the underlying strata, which increases the predisposition of the surfaces to mould growth once suitable conditions flourish [34–36]. Therefore it is strongly recommended that these surfaces are cleaned to avoid further degradation to the fabric.

## Conclusions

This study shows that the National Trust has instigated an effective maintenance programme that has secured stable indoor environmental conditions by conservation heating and cleaning that are vital to avoid mould growth, both airborne and on the surfaces. The previously reported mould problems concentrating especially on the vellum binders, back of the book presses, behind the picture frames and tapestries, which have quite possibly been exacerbated due to organic matter content, poor ventilation and rising damp, or external conditions leading to moisture enrichment, such as driven rain, seem to have been resolved. One critical area that emerges from this research however is around windows. Both the metal frame and stonework sills of the single glazed windows were found to have high surface mould concentrations. Because these are an intrinsic part of the architectural heritage of the Blickling Hall, and therefore cannot be replaced, it is of utmost importance that they are kept clean and free of surface water, in order to avoid conditions that do and can lead to surface mould.

In this study the air and surface benchmarks developed for UK residential properties (using the exact same testing protocol) were used to interpret the mould concentration values. The presence of abundant organic and vulnerable materials however might make museum environments more prone to mould growth under the same environmental conditions, and further research is needed to test the applicability of these benchmarks to heritage buildings.

## References

[1] Viitanen H (2010). Moisture and bio-deterioration risk of building materials and structures. J Build Phys.

[2] Horner WE, Barnes C, Codina R, Levetin E (2008). Guide for interpreting reports from inspections/investigations of indoor mold. J Allergy Clin Immunol.

[3] Hurraß J, Heinzow B, Aurbach U, Bergmann KC, Bufe A, Buzina W, Cornely OA, Engelhart S, Fischer G, Gabrio T, Heinz W, Herr CEW, Kleine-Tebbe J, Klimek L, Köberle M, Lichtnecker H, Lob-Corzilius T, Merget R, Mülleneisen N, Nowak D, Rabe U, Raulf M, Seidl HP, Steiß JO, Szewszyk R, Thomas P, Valtanen K, Wiesmüller GA (2017). Medical diagnostics for indoor mold exposure. Int J Hyg Environ Health.

[4] Aktas YD, D’Ayala D, Erkal A, Stephenson V (2015). Environmental performance assessment using monitoring and DVS testing. Proc Inst Civil Eng Eng History Herit.

[5] Sedlbauer K. Prediction of mould fungus formation on the surface of and inside building components, Ph.D. Thesis, Fraunhofer Institute for Building Physics, Germany. 2001.

[6] Abuku M, Jassen H, Roels S (2009). Impact of wind-driven rain on historic brick wall buildings in a moderately cold and humid climate: numerical analyses of mould growth risk, indoor climate and energy consumption. Energy Build.

[7] D’Ayala D, Aktas YD (2016). Moisture dynamics in the masonry fabric of historic buildings subjected to wind-driven rain and flooding. Build Environ.

[8] Brischke C, Rapp AO (2008). Dose–response relationships between wood moisture content, wood temperature and fungal decay determined for 23 European field test sites. Wood Sci Technol.

[9] Isaksson T, Thelandersson S, Ekstrand-Tobin A, Johansson P (2010). Critical conditions for onset of mould growth under varying climate conditions. Build Environ.

[10] Lankaster P, Brimblecombe P (2012). The impact of future climate on historic interiors. Sci Total Environ.

[11] Aktas YD, Ioannou I, Altamirano H, Reeslev M, May N, D’Ayala D, Canales M (2018). Surface and passive/active air mould sampling: a testing exercise in a North London housing estate. Sci Total Environ.

[12] Méheust D, Le Cann P, Reboux G, Millon L, Gangneux JP (2014). Indoor fungal contamination: health risks and measurement methods in hospitals, homes and workplaces. Crit Rev Microbiol.

[13] ASTM (2014). ASTM D7338-14: standard guide for fungal assessment in buildings.

[14] Reeslev M, Miller M. The Mycometer-test: a new rapid method for detection and quantification of mould in buildings. In: Proceedings of healthy buildings. Espoo, Finland; 2000. p. 589–590.

[15] Reeslev M, Miller M, Nielsen KF (2003). Quantifying mold biomass on gypsum board: comparison of ergosterol and Beta-*N*-acetylhexosaminidase as mold biomass parameters. Appl Environ Microbiol.

[16] Rylander R (2015). β-*N*-Acetylhexosaminidase (NAHA) as a marker of fungal cell biomass—storage stability and relation to β-Glucan. Int J Environ Monit Anal.

[17] Rylander R, Reeslev M, Hulander T (2010). Airborne enzyme measurements to detect indoor mould exposure. J Environ Monit.

[18] Chen Y, Aktas YD. Indoor Mould Testing of a Historical University Building: UCL Chadwick Building. London. In: Proceedings of 7th masters conference: people and buildings—network for comfort and energy use in buildings NCEUB. 2017.

[19] Stanley-Millson C, Newman J (1986). Blickling Hall: the building of a Jacobean mansion. Archit History.

[20] Adams M (1894). Blickling Hall, Norfolk: its drainage, water supply and other works. J R Inst Br Archit (RIBA).

[21] Blades N. Mitigating damage due to floods and moisture to heritage: The National Trust perspective. 2013. http://www.ucl.ac.uk/parnassus/international_parnassus_workshop/presentation_nigel_blades Accessed 18 June 2018.

[22] Strang T, Dawson J (1991). Controlling museum fungal problems.

[23] Guild S, MacDonald M (2007). Mould prevention and collection recovery.

[24] Bullock L (2009). Environmental control in National Trust properties. J Archit Conserv..

[25] Aktas YD, D’Ayala D, Blades N, Calnan C (2017). An assessment of moisture induced damage in Blickling Hall in Norfolk, England, via environmental monitoring. Herit Sci.

[26] Aktas YD, Altamirano H, Ioannou I, May N, D’Ayala D (2018). Indoor mould testing and benchmarking.

[27] Maunsell K (1952). Air-borne fungal spores before and after raising dust; sampling with sedimentation. Int Arch Allergy.

[28] Swaebly MA, Christensen CM (1952). Molds in house dust, furniture stuffing, and in the air within homes. J Allergy Clin Immunol.

[29] Liu Z, Li A, Hu Z, Sun H (2014). Study on the potential relationships between indoor culturable fungi, particle load and children respiratory health in Xi’an, China. Build Environ.

[30] Haas D, Galler H, Luxner J, Zarfel G, Buzina W, Friedl H, Marth E, Habib J, Reinthaler FF (2013). The concentrations of culturable microorganisms in relation to particulate matter in urban air. Atmos Environ.

[31] Kim KY, Park JB, Kim CN, Lee KJ (2006). Distribution of airborne fungi, particulate matter and carbon dioxide in Seoul metropolitan subway stations. J Prev Med Public Health.

[32] Pasanen A-L, Kalliokoski P, Pasanen P, Jantunen MJ, Nevalainen A (1991). Laboratory studies on the relationship between fungal growth and atmospheric temperature and humidity. Environ Int.

[33] Trust National (2015). Blickling Estate.

[34] Gaylarde CC, Morton LHG (1999). Deteriogenic biofilms on buildings and their control: a review. Biofouling.

[35] Grant C (1982). Fouling of terrestrial substrates by algae and implications for control: a review. Int Biodeterior Bull.

[36] Ortega-Calvo JJ, Ariño X, Hernandez-Marine M, Saiz-Jimenez C (1995). Factors affecting the weathering and colonization of monuments by phototropic microorganisms. Sci Total Environ.

